# Model selection for component network meta-analysis in connected and disconnected networks: a simulation study

**DOI:** 10.1186/s12874-023-01959-9

**Published:** 2023-06-14

**Authors:** Maria Petropoulou, Gerta Rücker, Stephanie Weibel, Peter Kranke, Guido Schwarzer

**Affiliations:** 1 Institute of Medical Biometry and Statistics, Faculty of Medicine and Medical Center – University of Freiburg, Stefan-Meier-Straße 26, 79104 Freiburg, Germany; 2 Department of Anaesthesiology, Intensive Care, Emergency and Pain Medicine, University Hospital Würzburg, Oberdürrbacher Straße 6, 97080 Würzburg, Germany

**Keywords:** Component network meta-analysis, Disconnected networks, Model selection, Multicomponent interventions, Simulation

## Abstract

**Background:**

Network meta-analysis (NMA) allows estimating and ranking the effects of several interventions for a clinical condition. Component network meta-analysis (CNMA) is an extension of NMA which considers the individual components of multicomponent interventions. CNMA allows to “reconnect” a disconnected network with common components in subnetworks. An additive CNMA assumes that component effects are additive. This assumption can be relaxed by including interaction terms in the CNMA.

**Methods:**

We evaluate a forward model selection strategy for component network meta-analysis to relax the additivity assumption that can be used in connected or disconnected networks. In addition, we describe a procedure to create disconnected networks in order to evaluate the properties of the model selection in connected and disconnected networks. We apply the methods to simulated data and a Cochrane review on interventions for postoperative nausea and vomiting in adults after general anaesthesia. Model performance is compared using average mean squared errors and coverage probabilities.

**Results:**

CNMA models provide good performance for connected networks and can be an alternative to standard NMA if additivity holds. For disconnected networks, we recommend to use additive CNMA only if strong clinical arguments for additivity exist.

**Conclusions:**

CNMA methods are feasible for connected networks but questionable for disconnected networks.

**Supplementary Information:**

The online version contains supplementary material available at 10.1186/s12874-023-01959-9.

## Background

**Fig. 1:**
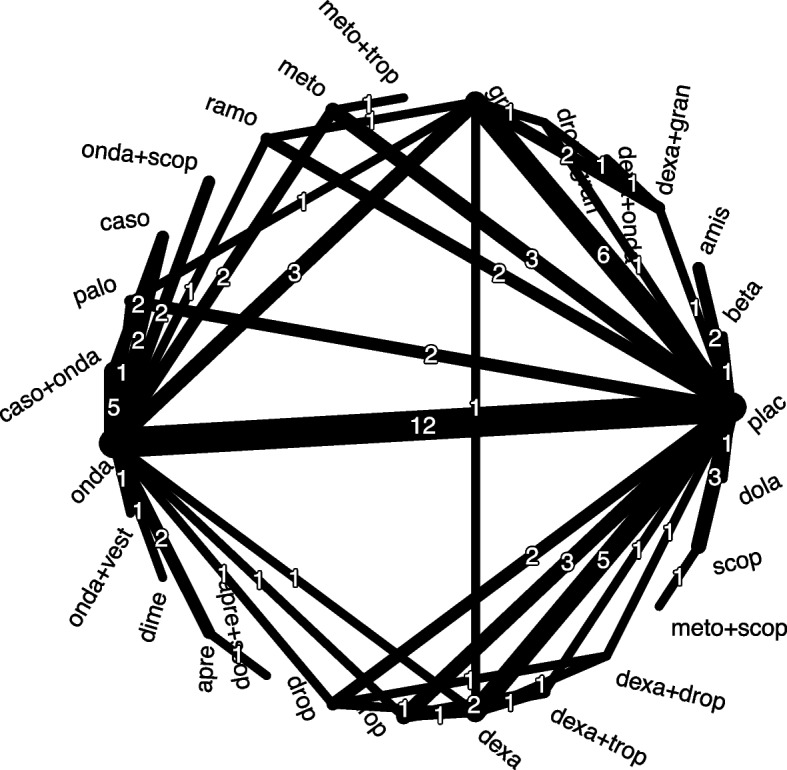
Network plot for the Cochrane data set (outcome: any adverse event). Line width corresponds to number of studies in direct comparisons. Abbreviations: amis: amisulpride; apre: aprepitant; beta: betamethasone; caso: casopitant; dexa:dexamethasone; dime: dimenhydrinate; dola: dolasetron; drop: droperidol; gran: granisetron; meto: metoclopramide; onda: ondansetron; palo: palonosetron; plac: placebo; ramo: ramosetron; scop: scopolamine; trop:tropisetron; vest: vestipitant. Numbers represent the number of studies

Standard network meta-analysis (NMA) synthesizes direct and indirect evidence of randomized controlled trials (RCTs) to estimate the effects of several competing interventions [1–3]. One requirement of standard NMA is that the network of interventions is connected. A network is connected if all interventions are either compared directly or via some intermediate interventions with any other intervention in the network. The network shown in Fig. 1 from a Cochrane review on interventions for postoperative nausea and vomiting in adults after general anaesthesia [4, 5] is connected. This network would no longer be connected if, for example, the two studies comparing aprepitant (apre) and ondansetron (onda) were not available. In this case, the comparison apre versus apre+scop would not be connected to the other interventions. In practice, many situations can lead to disconnected networks with two or more subnetworks when synthesizing evidence from RCTs [6].

Standard NMA is not possible in disconnected networks, instead, it can be simply replaced with separate NMA analyses for each of the subnetworks. Several alternative NMA methods have been proposed to deal with disconnected networks [7]. A theoretical framework of the Bayesian contrast-based model has recently been provided for disconnected networks [8]. Arm-based approaches have also been developed to analyse connected and disconnected networks [9–11]. Goring et al. [12] proposed the random baseline treatment effects NMA model in the Bayesian framework to accommodate disconnected networks, while Béliveau et al. [6] conducted a case study to evaluate the performance of the random baseline treatment effects in disconnected networks. Mixture models of RCTs and observational studies can “reconnect” disconnected networks using matching-adjusted indirect comparisons [13–18] or hierarchical models [19]. Linking disconnected networks can also be addressed through dose-response relationships [20] or component NMA [21, 22] if subnetworks share common intervention doses or components—it is worth noting that this is not the main purpose of dose-response or component NMA.

Many healthcare interventions consist of multiple, possibly interacting, components. Several meta-analytical models address the effects of such complex interventions [23]. Component network meta-analysis (CNMA), a generalization of standard NMA, estimates the effects of components of complex interventions and has been introduced under both Bayesian and frequentist framework [21, 22]. Wigle and Béliveau [24] showed that the CNMA model by Welton et al. [21] is more restrictive as all component effects are modeled relative to a so called anchor intervention which is not required in the model by Rücker et al. [22]. Accordingly, Wigle and Béliveau [24] introduced a Bayesian CNMA model similar to the frequentist model [22] and conducted a simulation study comparing their newly proposed and existing CNMA models in connected networks. Efthimiou et al. [25] describe several extensions of the Bayesian CNMA model including methods for individual participant data.

An additive CNMA model assumes that the effect of any combination of components is the additive sum of their components, known as the additivity assumption. This assumption can be relaxed by adding interaction terms allowing components of complex interventions to interact, either synergistically or antagonistically. Adding interactions might improve the goodness of fit, but also decrease the network connectivity. Accordingly, we conduct CNMA model selection with the aim to find a model with a reasonable balance between the goodness of fit and connectivity (selected CNMA). The forward model selection for CNMA models, which has recently been developed [26], starts with an additive CNMA model and adds interaction terms until some goodness of fit criterion has been reached. We call the additive CNMA model sparse as it contains the smallest number of parameters. Additional parameters have to be estimated in interaction CNMA models which we thus call richer models.

At the moment, there is no established guidance concerning which CNMA approach (a sparse or rich version) fits best under different circumstances for connected or disconnected networks. Therefore, we conducted a comprehensive simulation study based on the CNMA models by Rücker et al. [22] to investigate the performance of the forward selection approach [26] in connected and disconnected networks. In the simulations, we assumed that additivity either holds or is mildly or strongly violated for one combination. Starting with a connected network, we artificially constructed disconnected networks and implemented the forward selection strategy to select the best CNMA model for each disconnected network.

For connected networks, we compared mean square errors and coverage probabilities of the selected CNMA model with those of the standard NMA and additive CNMA model. We investigated the circumstances under which a sparse (e.g., additive) or a richer (e.g, selection-based) CNMA model is preferable to the standard NMA. For disconnected networks, we compared the results of the additive and selected CNMA model. We applied CNMA model selection also to data of a Cochrane review on postoperative nausea and vomiting [4, 5].

The paper is organized as follows: The Cochrane data set used as an example is briefly described in the next section. In the Methods section, we describe the (C)NMA models evaluating the effects of complex interventions and the CNMA model selection method. In the following section, we summarize the design of our simulation study. Afterwards, we outline the results of our simulation study and we apply our methods to the Cochrane data set. In the Discussion section, we provide the main findings of the study and, finally, we end up with the conclusions.

## Data set on adverse events in adults after general anaesthesia

We illustrate the CNMA model selection process using a published Cochrane review of 585 RCTs that compares complex interventions for postoperative nausea and vomiting in adults after general anaesthesia [4, 5]. Here we consider the outcome of any adverse event with the risk ratio as effect measure. The outcome was available in 61 RCTs of which four RCTs without any adverse event were excluded due to an inestimable risk ratio. The remaining 57 RCTs comprise 44 studies comparing two interventions (two-arm) as well as 11 three-arm and 2 four-arm studies.

In total, 27 interventions are compared, including 15 single interventions / components (e.g., ondansetron (onda), scopolamine (scop)), 11 complex interventions (e.g., ondansetron plus scopolamine (onda+scop)), and placebo (Fig. 1). A total of 16 interventions are compared directly with placebo (e.g., dolasetron (dola) versus placebo). The interventions contain 17 components (including placebo), one component (vest) was only evaluated in a combination: onda + vest. As Fig. 1 shows, the network of interventions is connected.

## Methods

### Standard NMA

Standard NMA assumes that each (single or combined) intervention has its own effect which is represented as a node in the network. We follow the frequentist approach introduced by Rücker et al. [27]. Suppose we have data consisting of *m* pairwise comparisons with *n* interventions, and let $\varvec{\theta }$ represent the *n* intervention-based (true) responses. Let $\textbf{d}= (d_{1}, d_{2},..., d_{m})$ be the observed (relative) intervention effects with the associated standard error $\text {SE} (d_j)$ for each comparison $j = 1, \dots , m$. Assuming a common between-study variance (heterogeneity $\tau ^2$) across the pairwise comparisons, the random-effects network meta-analysis model is

$$\begin{aligned} \textbf{d} = \textbf{X}\varvec{\theta } + \varvec{\mu } + \varvec{\epsilon }, \varvec{\epsilon } \sim \mathcal {N}(\textbf{0},\varvec{\Sigma }), \varvec{\mu }\sim \mathcal {N}(\textbf{0},\varvec{\Delta }) \end{aligned}$$

where $\textbf{X}$ is the $m \times n$ design matrix describing the network structure, $\varvec{\Sigma }$ is the within-study variance-covariance matrix, and $\varvec{\Delta }$ is the between-study variance-covariance matrix. Let $\textbf{W}$ be a diagonal $m \times m$ weight matrix with a vector of weights on its diagonal. The weight for each two-arm study is the inverse of the sum of the within- and between-study variance. For multi-arm studies, the weights are assumed to be adjusted as described in [28]. We can write the standard NMA model briefly ${\varvec{\delta }} = \textbf{X}\varvec{\theta }$ where ${\varvec{\delta }}$ denotes the vector of true relative intervention effects which is estimated using weighted least squares regression ($\hat{\varvec{\delta }}$). Cochran’s *Q* statistic is given by $Q = (\textbf{d} - \hat{\varvec{\delta }}) ^\top \textbf{W} (\textbf{d} - \hat{\varvec{\delta }})$ which follows a chi-square distribution with degrees of freedom ${df} = n_a - k - (n - 1)$, where $n_a$ is the total number of intervention arms and *k* is the number of studies. More details for the model can be found in [27] and [28]. In the challenging case of disconnected networks, standard NMA is not possible, but it can be replaced with separate NMA analyses for each of the subnetworks.

### Additive CNMA

The sparse additive CNMA model assumes that the effect of each combined intervention is the additive sum of the effects of its components, that is, equal components cancel out in pairwise comparisons [21, 22]. Let the number of components be *c*. Having the data consisting of *m* pairwise comparisons with *n* interventions, the design matrix of the additive CNMA model is the $m \times c$ matrix given by $\textbf{X}_{a} =\textbf{B}\textbf{C}$ , where $\textbf{B}$ is the $m \times n$ design matrix describing which interventions are compared in each pairwise comparison, and $\textbf{C}$ is the $n \times c$ combination matrix describing the information on how the *n* interventions are composed of the *c* components [22]. The additive CNMA model is given by

1 $$\begin{aligned} {\varvec{\delta }}_{a} = \textbf{X}_{a} \varvec{\beta } = \textbf{B} \textbf{C} \varvec{\beta } = \textbf{B} \varvec{\theta }_{a} \end{aligned}$$

where ${\varvec{\delta }}_{a} \in \mathbb{R}^{m}$ is the vector of true relative intervention effects, $\varvec{\beta } \in \mathbb{R}^{c}$ a parameter vector of length *c*, representing the component effects, and $\varvec{\theta }_{a} = \textbf{C} \varvec{\beta } \in \mathbb{R}^{n}$ a vector of length *n*, representing the intervention effects.

### Interaction CNMA

The interaction CNMA model is an extension of the additive CNMA model [21, 22]. The model assumes an interaction between two or more observed components (antagonistically or synergistically) and therefore the combination of components provides larger or smaller effects than the sum of their effects, respectively. The interactions of interest can be added as additional columns to the combination matrix $\textbf{C}$ [22]. For *l* interactions, the combination matrix $\textbf{C}_{int}$ is of dimension $n \times (c+l)$. An interaction CNMA model is implemented in complete analogy to the additive CNMA model. Therefore, having the design matrix $\textbf{X}_{int} =\textbf{B}\textbf{C}_{int}$, the interaction CNMA model is given by

2 $$\begin{aligned} {\varvec{\delta }}_{int} = \textbf{X}_{int} \varvec{\beta }_{int} = \textbf{B} \textbf{C}_{int} \varvec{\beta }_{int} = \textbf{B} \varvec{\theta }_{int} \end{aligned}$$

where ${\varvec{\delta }}_{int} \in \mathbb{R}^{m}$ is the vector of true relative intervention effects, $\varvec{\beta }_{int}$ a parameter vector of length $c+l$, representing the component and interaction effects, and $\varvec{\theta }_{int} = \textbf{C}_{int}\varvec{\beta }_{int} \in \mathbb{R}^{n}$ a vector of length *n*, representing the intervention effects.

$\hat{\varvec{\delta }}_{a}$ from the additive and $\hat{\varvec{\delta }}_{int}$ from the interaction model are estimated using weighted least squares regression. Details on the estimation and the multivariate version of Cochran’s Q for CNMA models can be found in Rücker et al. [22].

### CNMA model selection

Table 1 illustrates the interrelation between goodness of fit and connectivity in (C)NMA models for connected and disconnected networks with complex interventions. For connected networks, the standard NMA model is the richest model as each complex intervention corresponding to a model parameter. The other extreme is an additive CNMA model with unique components as parameters. The number of model parameters of interaction CNMA models lies between these extremes depending on the number of interaction terms. Note, we describe an algorithm to identify inestimable interactions in the supplement as only estimable interactions should be considered in interaction CNMA models.

**Table 1:** (C)NMA models for (dis-)connected networks. $n_{c} =$ number of subnetworks (connectivity components), $n_{a} =$ number of intervention arms, $k =$ number of studies, $n=$ number of interventions, $r=$ rank of the design matrx of additive CNMA model, $\nearrow :$ increasing number of interactions in CNMA model, $\searrow :$ decreasing number of interactions in CNMA model

			Connected network	Disconnected network
	CNMA models		NMA	Separate NMAs for each subnetwork
	Additive model	Interaction models		
No. of interactions	none	1, 2, 3, ...	all observed	all observed
Model fit	often poor fit	$\nearrow$	often good fit	maximal fit
*Q*		$\searrow$		minimal $Q=\sum _{i = 1}^{n_c}Q_i$
Connectivity	good connectivity	$\searrow$	poor connectivity	minimal connectivity
*df*	maximal ${df}=n_a - k - r$	$\searrow$	${df}=n_a - k - (n - 1)$	minimal ${df}=n_a - k - (n - n_c)$

The standard NMA model has the smallest *Q* statistic and degrees of freedom (*df*) as it fits the data better than the other models. On the other hand, the additive model is the most parsimonious (sparse) model, i.e., it has the largest *Q* and *df* values. Adding estimable interactions to a CNMA model typically decreases *Q*, thus improving the goodness of fit, but also decreases the degrees of freedom and, most notably, may decrease the network connectivity (${df}\searrow$)(Table 1). An interaction CNMA model with all estimable interactions is equivalent to a standard NMA model, i.e., has the same *Q* and *df* values [22].

Rücker et al. [26] introduced a model selection procedure for CNMA models with the aim to find the CNMA model with a reasonable balance between the goodness of fit and connectivity. There are two possible directions: forward selection and backward selection which either add or remove estimable interaction terms.

Forward CNMA model selection starts with the additive (sparse) CNMA model and is moving forward to richer models. During the selection process, estimable interactions are gradually added to the model until a stopping criterion is fulfilled [26]. We use the Akaike Information criterion (AIC) as stopping criterion [29], i.e., the selection process stops if all *p*-values of the remaining estimable interactions are above 0.157.

Backward CNMA model selection should start with an interaction CNMA model having the same value for Cochran’s *Q* as the standard (rich) NMA model. During the selection process, one interaction term is removed from the model in each step until a stopping criterion is fulfilled [26]. One difficulty in connected networks is to determine a sufficient number of estimable interactions to get the same value for Cochran’s *Q* as the standard NMA model. In disconnected networks, a standard NMA is impossible and in this case, additivity can be assumed for just one component that is common to all subnetworks [26]. Therefore, we may start with ‘separating’ one component which is common to all subnetworks [26]. Such a model usually provides a good fit, it may even provide the minimum *Q*, given by the sum over all *Qs* from the subnetworks. However, it provides only a very loose connection between the subnetworks and is associated with small *df* (Table 1). For this reason, under all models giving the same model fit, we prefer those with greater connectivity than those with smaller connectivity. In other words, we prefer sparse models to rich models.

### Construction of disconnected networks

As described in the previous section, CNMA model selection can be applied to connected and disconnected networks. To evaluate model selection in disconnected networks, we artificially constructed disconnected networks in the Cochrane data and the simulation study following an approach similar to Béliveau et al. [6].

Having a connected network with *n* interventions, we constructed disconnected networks with two or more separate subnetworks under the constraint of retaining all *n* interventions. We started by constructing a *minimal set* of interventions which will be part of one subnetwork:

1. Select one of the *n* interventions as a reference intervention which is the nucleus of the minimal set.
2. All interventions that are only compared directly with the reference are added to the minimal set.
3. All interventions of a branch without any loops connected to the reference are added to the minimal set.
4. All interventions of a multi-arm study are added to the minimal set if any arm in this multi-arm study is only directly compared with the reference.
5. Any remaining intervention in the network that is only compared to an intervention in the minimal set is also added.

All interventions of multi-arm studies identified in step 4 must be added to the minimal set as these interventions could not be included in a different subnetwork and ignoring the multi-arm study would result in a network with fewer than *n* interventions. Step 5 is applied recursively until all relevant interventions are added to the minimal set.

Interventions included in the minimal set are the core of a so called *main subnetwork* containing the reference. Additional interventions could be added to the main subnetwork as long as at least one other subnetwork exists. In well-connected networks, the minimal set can consist of the reference intervention only. In this case at least one additional intervention has to be added to the minimal set to form the main subnetwork.

All interventions not included in the main subnetwork must be part of an *auxiliary subnetwork*. A disconnected network is constructed by removing all studies comparing interventions from different subnetworks. Different disconnected networks can be constructed by adding different interventions to the minimal set which results in different main and auxiliary subnetworks.

The minimal set must be defined / constructed by the user, however, we wrote an R script to identify all disconnected networks for a given minimal set (see R function disconnect_additional on https://zenodo.org/badge/latestdoi/546041022).

#### Construction of minimal set in Cochrane data set

The minimal set for the Cochrane data with placebo as reference (step 1) consists of 14 interventions in addition to placebo (table in Additional file 1). The following interventions are only connected to placebo and are thus added to the minimal set in step 2: amis, beta, dola (see Fig. 1). The interventions scop and meto + scop constitute a branch without loops connected to placebo (step 3). The interventions of the two four-arm studies (dexa, drop, dexa + drop, placebo; gran, dexa + gran, drop + gran, placebo) are added to the minimal set as dexa + drop and dexa + gran are only evaluated in the four-arm studies (step 4). Also, the interventions of the three-arm study comparing dexa, dexa + trop and placebo must be part of the minimal set due to the intervention dexa + trop (step 4). The intervention dexa + onda is added to the minimal set as this intervention is only compared to dexa + gran which is already part of the minimal set (step 5). Finally, intervention trop is added to the minimal set as all comparisons with trop contain either placebo or an intervention already in the minimal set (step 5).

## Simulation design

**Fig. 2:**
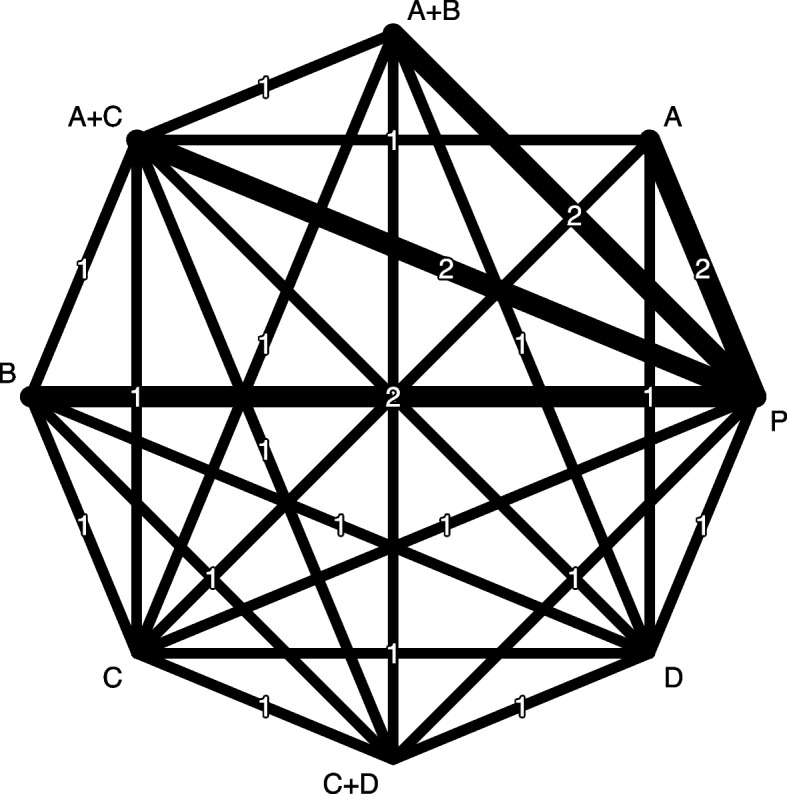
Network plot of simulated network. Line width corresponds to numbers of studies in direct comparisons

We simulated data for a network of two-arm studies with eight interventions ($n = 8$): four single treatments (*A*, *B*, *C*, *D*), three combinations $(A+B, A+C, C+D)$ and placebo *P*. The network is well-connected, however, omits the direct comparisons *A* versus *B*, *A* versus $A+B$ and *A* versus $C+D$ (Fig. 2). We assumed two studies directly comparing each of interventions $A, B, A+B, A+C$ with placebo (which was chosen as the reference) and only a single study for other comparisons. We generated arm-level dichotomous outcome data with odds ratio as effect measure and we assumed a common heterogeneity variance $\tau ^2$ for all pairwise comparisons. Non-equal true relative effects (${\varvec{\delta }}=\log (\text {OR})$) were set with $e^{\delta _{A,P}} = 1.40$, $e^{\delta _{B,P}} = 1.20$, $e^{\delta _{C,P}} = 2.30$ and $e^{\delta _{D,P}} = 1.50$. A summary of all simulation parameters is given in Table 2.

**Table 2:** Overview of simulated scenarios

**Network geometry**	Well-connected network
Studies/Pairwise comparisons	$k=28$
**Interventions**	$n=8$
	single: *A*, *B*, *C*, *D*,
	combined: $A+B,A+C,C+D,$
	reference: placebo *P*
**Additivity assumption on relative intervention effects**	
Scenario A: Additive effects	$\delta _{A+B,P} = \delta _{A,P} + \delta _{B,P}$
	$$\delta _{A+C,P} = \delta _{A,P} + \delta _{C,P}$$
	$$\delta _{C+D,P} = \delta _{C,P} + \delta _{D,P}$$
Scenario B: Mild violation of additivity assumption	
B1: combined intervention $A+B$	$\delta _{A+B,P} = \delta _{A,P} + \delta _{B,P} + \lambda _{AB}, e^{\lambda _{AB}} = 1.5$
B2: combined intervention $C+D$	$\delta _{C+D,P} = \delta _{C,P} + \delta _{D,P} + \lambda _{CD}, e^{\lambda _{CD}} = 1.5$
Scenario C: Strong violation of additivity assumption	
C1: combined intervention $A+B$	$\delta _{A+B,P} = \delta _{A,P} + \delta _{B,P} + \lambda _{AB}$, $e^{\lambda _{AB}} = 2.0$
C2: combined intervention $C+D$	$\delta _{C+D,P} = \delta _{C,P} + \delta _{D,P} + \lambda _{CD}$, $e^{\lambda _{CD}} = 2.0$
**Heterogeneity**	
No heterogeneity	$\tau ^2=0.00$
Low heterogeneity	$\tau ^2=0.01$
Moderate heterogeneity	$\tau ^2=0.10$
**Inconsistency**	No inconsistency
**Other simulation parameters**	
True relative intervention effects	$e^{\delta _{A,P}}= 1.40$, $e^{\delta _{B,P}} = 1.20$, $e^{\delta _{C,P}}= 2.30$, and $e^{\delta _{D,P}}= 1.50$
Baseline probability	$p_{P}=0.1$
Patients per study arm	$n_i\sim \mathcal {U}(50, 200)$
Iterations	$M=1000$

Starting with the connected network in Fig. 2, we artificially constructed disconnected networks differing in network geometry, number of included studies, and pairwise comparisons. The forward model selection strategy was implemented and the selection-based CNMA model was chosen for each disconnected network.

Following Thorlund and Mills [30], we were interested in three scenarios for intervention effects:

(A) *All relative intervention effects are additive* For any two interventions, say *A* and *B*, the relative effect of the intervention $A+B$ comprising two components *A* and *B* versus intervention *P* is the additive sum of the relative intervention effect of *A* versus *P* and the relative intervention effect of *B* versus *P*:
  $$\begin{aligned} \delta _{A+B,P} = \delta _{A,P} + \delta _{B,P} \end{aligned}$$
(B) *The additivity assumption for one intervention is mildly violated with a relevant synergistic interaction* Assuming an interaction ratio $IR_{AB}$ between interventions *A* and *B*, the relative intervention effect of $A+B$ versus *P* is
  $$\begin{aligned} \delta _{A+B,P} = \delta _{A,P} + \delta _{B,P} + \lambda _{AB} \end{aligned}$$
  with $\lambda _{AB} = \log (IR_{AB})$. Following Thorlund and Mills [30], we set $IR_{AB}=1.5$.
(C) *The additivity assumption for one intervention is strongly violated with a relevant synergistic interaction*. The same model equation is used as for mild violation of additivity, however, the interaction ratio is larger in this scenario, $IR_{AB}=2.0$.

Scenario A assumes additivity for all combined interventions in the network, i.e., $A+B, A+C$, and $C+D$. Two variants of scenarios B and C were considered. First, we assumed mild or strong violation of additive effects for the combined intervention $A+B$ which are labelled scenarios B1 and C1. Second, we assumed mild or strong violation of additive effects for the combined intervention $C+D$ which are labelled scenario B2 and C2. Each scenario was repeated $M=1000$ times.

### Create disconnected networks

In our simulation study, we started by simulating connected networks and afterwards, we artificially constructed all possible disconnected networks as described above. The well-connected network in our simulation study does not have any intervention only connected to the reference *P*. Accordingly, the smallest main subnetwork in the simulation study is any single active intervention vs *P*. Additional interventions can be added to this small main subnetwork. In each simulation run, we randomly selected one disconnected network from the set of possible disconnected networks.

### Generation of simulated data

The generation of binary data was similar to Kiefer et al. [31]. For each study $i = 1, \dots , k$, we generated study-specific log-odds ratios $d_{iX,Y}$ from a normal distribution with mean $d_{X,Y}$ and between-study variance $\tau ^2$ with $X,Y \in \{A,B,C,D,A+B,A+C,C+D,P\}$, $X \ne Y$, representing the set of possible interventions. The baseline probability for placebo was set to 0.1. Study-specific probabilities for intervention $t\in \{A,B,C,D,A+B,A+C,C+D\}$ were calculated by

$$p_{it}= \frac{0.1\exp (d_{it,P})}{1-0.1(1-\exp (d_{it,P}))}$$

with log-odds ratios $d_{it,P}$.

For each study, we generated equal arm sample sizes $n_i$ from a discrete uniform distribution assuming values from 50 to 200. For study arms 1 and 2, we generated the number of events $e_{it_1}$ and $e_{it_2}$, $t_1, t_2 \in \{A,B,C,D,A+B,A+C,C+D, P\}, t_1 \ne t_2$, randomly from a binomial distribution with parameters $n_i$ and $p_{it_1}$ or $p_{it_2}$. If the simulated number of events $e_{it_1}$ or $e_{it_2}$ was zero, we added the value 0.5 to both event numbers.

### Simulation performance

We first calculated the mean squared error (MSE) and the coverage probability (CP) of the relative intervention effect, for each pair of the seven active interventions and placebo. Then, we calculated the average MSE and CP to summarize the properties of (C)NMA model fit. For each setting (connected or disconnected network, scenarios A to C2) and value of $\tau ^2$, we calculated the average MSE as

$$\begin{aligned} \text {MSE} = \frac{1}{M} \sum _{m=1}^M \frac{1}{7} \left\| \hat{\varvec{\delta }}_{m} - {\varvec{\delta }} \right\| ^2 \end{aligned}$$

where $\hat{\varvec{\delta }}_m$ denotes the vector with estimated relative intervention effects in iteration *m* and ${\varvec{\delta }}$ denotes the corresponding true effects. The division by 7 refers to the seven baseline parameters in this network.

The average coverage probability was similarly defined

$$\begin{aligned} \text {CP} = \frac{1}{M} \sum _{m=1}^M \frac{1}{7} \ \left\| {\textbf {1}}_{\hat{\varvec{\delta }}_{m, L} \, \le \, {\varvec{\delta }} \, \le \, \hat{\varvec{\delta }}_{m, U}} \right\| \end{aligned}$$

where $\hat{\varvec{\delta }}_{m, L}$ and $\hat{\varvec{\delta }}_{m, U}$ are vectors with lower and upper 95% confidence limits calculated in iteration *m* and $\textbf{1}$ is a vector of indicator functions.

### Software implementation

The simulation study was performed using the statistical software R [32]. (C)NMA models were fitted with R package **netmeta** using function netcomb for connected networks and discomb for disconnected networks [33].

## Results

For each scenario and value of $\tau ^2$, 1000 connected and disconnected networks were simulated and the CNMA model selection was performed.

### CNMA model selection for simulated connected networks

Table 3 summarizes simulation results for connected networks. The percentage of networks rejecting the additivity assumption increases from 3.5% to 18.8% with increasing $\tau ^2$ when assuming additive effects (scenario A). Accordingly, the test for additivity is too conservative for $\tau ^2 = 0$ and too liberal for $\tau ^2 = 0.1$. Moreover, the power to detect the violation of additivity is low for scenario B (25.7% to 42.0%) and moderate for scenario C (65.6% to 73.8%). CNMA model selection worked best for no heterogeneity. In general, the number of simulations selecting the correct model is decreasing with increasing heterogeneity. If the additivity assumption holds (scenario A), the correct additive CNMA model was only selected in the majority of simulations in the CNMA model selection for no or low heterogeneity (69.3% and 65.2%). For moderate heterogeneity, less than 50% of simulations selected the additive CNMA model. For mild violation of the additivity assumption (scenario B1 and B2), the correct interaction CNMA model was selected in less than 50% of simulations (34.6% to 45.0%). For strong violation of additivity (scenarios C1 and C2), the correct interaction CNMA model was always selected in the majority of simulations (54.6% to 74.6%).

**Table 3:** Selected CNMA models in 1000 simulations of connected networks

Scenario	$n_{diff}$	Additive CNMA	CNMA with one 2-way interaction			CNMA with two 2-way interactions		
			$A\!*\!B$	$A\!*\!C$	$C\!*\!D$	$A\!*\!B$+$C\!*\!D$	$A\!*\!B$+$A\!*\!C$	$A\!*\!C$+$C\!*\!D$
No heterogeneity ($\tau ^2=0.00$)								
A	35	**693**	119	94	84	3	2	5
B1	257	324	**436**	148	61	16^a^	12^a^	3
B2	298	325	45	154	**436**	17^a^	8	15^a^
C1	687	61	**746**	99	17	34^a^	28^a^	15
C2	732	51	13	104	**742**	38^a^	7	45^a^
Low heterogeneity ($\tau ^2=0.01$)								
A	52	**652**	136	98	108	3	1	2
B1	281	337	**426**	136	59	16^a^	18^a^	8
B2	284	317	49	133	**450**	25^a^	10	16^a^
C1	676	75	**704**	109	24	29^a^	47^a^	12
C2	738	70	13	98	**716**	45^a^	7	51^a^
Moderate heterogeneity ($\tau ^2=0.10$)								
A	188	**458**	188	142	179	14	13	6
B1	392	277	**346**	178	132	34^a^	17^a^	16
B2	420	262	122	195	**356**	28^a^	13	24^a^
C1	656	117	**550**	135	53	65^a^	64^a^	16
C2	702	93	48	161	**546**	65^a^	18	69^a^

The correctly chosen model is printed in **bold**

^a^Combination of two 2-way interactions includes the correct interaction

$n_{diff}$: number of networks rejecting the additivity assumption with significant *p*-value for $Q_{diff}$ ($p < 0.05$), where $Q_{diff}$ is the *Q* statistic for the difference between additive CNMA and standard NMA model

**Fig. 3:**
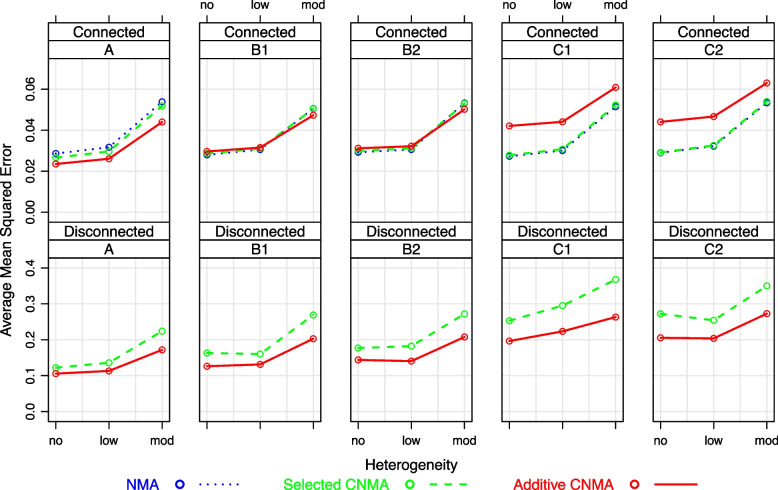
Average mean squared errors for simulated connected networks (top panel) and disconnected networks (bottom panel). Different scales are used on the y-axis due to the large differences in MSEs for connected and disconnected networks

Fig. 3, top panel, provides the average MSEs for the simulated connected networks. Average MSEs get larger with increasing heterogeneity for all models. Overall, the selected CNMA and standard NMA have very similar average MSEs. Under scenario A, average MSEs of the additive CNMA model is slightly smaller than the average MSEs for selected CNMA and standard NMA. All models perform on average equally for mild violation of the additivity assumption (scenarios B1 and B2), while selected CNMA and standard NMA models perform better for a large violation of the additivity assumption (scenarios C1 and C2). The figure in Additional file 2 shows that MSEs of the relative intervention effects are comparable for selected CNMA and standard NMA model, while the additive CNMA model has inferior results for several intervention estimates for a strong violation of the additivity assumption (e.g., *B* vs *P*, *C* vs *P* and $A+B$ (scenario C1) and *A* vs *P* and *D* vs *P* (scenario C2)).

**Fig. 4:**
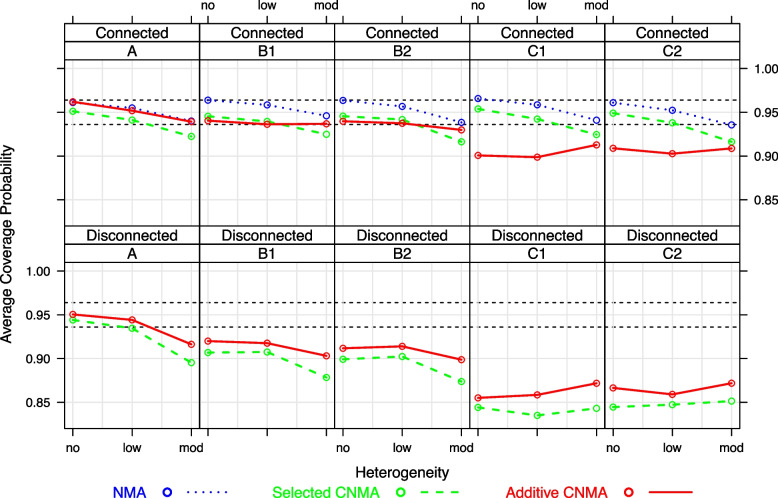
Average coverage probabilities for simulated connected networks (top panel) and disconnected networks (bottom panel). Dashed lines denote the 95% Monte-Carlo limits $(L,U)=[0.936; 0.964]$ for CP

Figure 4, top panel, provides the average coverage probabilities for the simulated connected networks. The average CP is decreasing with increasing heterogeneity for all models when additivity holds (scenario A). Average CPs are very similar for standard NMA and additive CNMA. Average CPs lie within the 95% Monte-Carlo limits, with exception of the selected CNMA model for moderate heterogeneity. For mild and strong violation of the additivity assumption, only the average CP of the standard NMA model always falls within the 95% Monte-Carlo limits for any value of $\tau ^2$. Average CPs for the additive model are well below the lower Monte-Carlo limit for scenarios C1 and C2. The figure in Additional file 3 shows that CPs of the relative intervention effects are in general somewhat smaller for the selected CNMA model compared to the standard NMA model, however, not dramatically different. For the additive CNMA model, CPs are much smaller than the Monte-Carlo limits for the intervention estimates with large MSE values.

### CNMA model selection for simulated disconnected networks

Table 4 summarizes simulation results for disconnected networks. In comparison to connected networks, no test of the additivity assumption is available, as a standard NMA model cannot be estimated in a disconnected network. Despite using the rather liberal Akaike criterion, the additive CNMA model is selected in the majority of simulations under mild violation of additivity (58.3% to 70.4%) and in a large proportion of simulations under strong violation of the additivity assumption (46.2% to 51.5%). The correct interaction CNMA model is only selected in 12.0% to 15.2% under scenarios B1 and B2 and 21.6% to 26.3% under scenarios C1 and C2. Therefore, CNMA model selection did not work well in our simulated disconnected networks.

**Table 4:** Selected CNMA models in 1000 simulations of disconnected networks

Scenario	Additive CNMA	CNMA with one 2-way interaction			CNMA with two 2-way interactions		
		$A\!*\!B$	$A\!*\!C$	$C\!*\!D$	$A\!*\!B$+$C\!*\!D$	$A\!*\!B$+$A\!*\!C$	$A\!*\!C$+$C\!*\!D$
No heterogeneity ($\tau ^2=0.00$)							
A	**834**	52	55	57	1	0	1
B1	698	**121**	120	57	1^a^	1^a^	2
B2	693	79	93	**131**	1^a^	3	0^a^
C1	511	**227**	158	98	2^a^	2^a^	2
C2	469	107	179	**237**	2^a^	2	4^a^
Low heterogeneity ($\tau ^2=0.01$)							
A	**809**	51	72	68	0	0	0
B1	704	**120**	94	79	1^a^	1^a^	1
B2	677	83	99	**138**	1^a^	1	1^a^
C1	515	**216**	151	111	6^a^	0^a^	1
C2	480	101	150	**263**	1^a^	0	5^a^
Moderate heterogeneity ($\tau ^2=0.10$)							
A	**664**	116	110	103	3	2	2
B1	598	**148**	131	115	5^a^	1^a^	2
B2	583	137	119	**152**	2^a^	4	3^a^
C1	462	**220**	171	137	6^a^	3^a^	1
C2	485	130	154	**216**	8^a^	4	3^a^

The correctly chosen model is printed in bold

^a^ The chosen combination of two 2-way interactions includes the correct interaction

Figure 3, bottom panel, shows that average MSEs for disconnected networks are much larger than for connected networks for all scenarios. Furthermore, average CPs for disconnected networks only fall into the 95% Monte-Carlo limits for scenario A in case of no or low heterogeneity (Fig. 4, bottom panel). Average MSEs and CPs for the selected CNMA are always worse than for the additive CNMA, even under scenarios C1 and C2 with strong violation of the additivity assumption (Figs. 3 and 4, bottom panels). This general pattern is also observed for MSEs and CPs of the relative intervention effects (figures in Additional files 4 and 5).

### CNMA model selection in Cochrane review on postoperative nausea and vomiting

We applied the forward CNMA model selection procedure to this connected network. Results for standard NMA, additive CNMA and selected CNMA model can be found in Additional file 6. We used the strategy described in the Methods section to construct disconnected networks. The minimal set for the Cochrane data with placebo as reference consists of 15 interventions including placebo (Additional file 1). We removed 20 studies with 34 pairwise comparisons from the data set in order to separate the minimal set (with 15 interventions, 21 studies, 35 pairwise comparisons) from the auxiliary network (12 interventions, 16 studies, 20 pairwise comparisons). We identified 18 additional disconnected networks by adding interventions to the minimal set. The disconnected networks differ substantially in the number of included studies and pairwise comparisons. In Table 5 and the figure in Additional file 7, disconnected networks were sorted by decreasing number of pairwise comparisons, studies, and pairwise comparisons in the main subnetwork, with the largest network containing 55 studies and 87 pairwise comparisons and the smallest network only 35 studies with 53 pairwise comparisons distributed over three subnetworks.

**Table 5:** Results of CNMA model selection for disconnected networks in Cochrane data set with number of studies *k*, pairwise comparisons *m*, and number of subnetworks *s*

Network	*k*	*m*	*s*	First interaction	Second interaction	Third interaction	Fourth interaction
Connected	57	89	1	onda$*$scop	apre$*$scop	meto$*$trop	
Disconnected 1	55	87	2	onda$*$scop	apre$*$scop	meto$*$trop	
Disconnected 2	53	83	2	onda$*$scop	apre$*$scop	meto$*$trop	
Disconnected 3	51	81	3	onda$*$scop	apre$*$scop	meto$*$trop	
Disconnected 4	41	61	2	onda$*$scop	dexa$*$trop	dexa$*$gran	
Disconnected 5	40	60	2	onda$*$scop	dexa$*$trop	dexa$*$gran	dexa$*$drop
Disconnected 6	39	59	3	onda$*$scop	dexa$*$trop	dexa$*$gran	
Disconnected 7	39	59	2	onda$*$scop	dexa$*$trop	dexa$*$gran	
Disconnected 8	40	58	2	onda$*$scop	dexa$*$trop	dexa$*$gran	
Disconnected 9	38	58	3	onda$*$scop	dexa$*$trop	dexa$*$gran	dexa$*$drop
Disconnected 10	38	58	2	onda$*$scop	dexa$*$trop	dexa$*$gran	dexa$*$drop
Disconnected 11	39	57	2	onda$*$scop	dexa$*$trop	dexa$*$gran	dexa$*$drop
Disconnected 12	37	57	3	onda$*$scop	dexa$*$trop	dexa$*$gran	
Disconnected 13	38	56	3	onda$*$scop	dexa$*$trop	dexa$*$gran	
Disconnected 14	38	56	2	onda$*$scop	dexa$*$trop	dexa$*$gran	
Disconnected 15	36	56	3	onda$*$scop	dexa$*$trop	dexa$*$gran	dexa$*$drop
Disconnected 16	37	55	3	onda$*$scop	dexa$*$trop	dexa$*$gran	dexa$*$drop
Disconnected 17	37	55	2	onda$*$scop	dexa$*$trop	dexa$*$gran	dexa$*$drop
Disconnected 18	36	54	3	onda$*$scop	dexa$*$trop	dexa$*$gran	
Disconnected 19	35	53	3	onda$*$scop	dexa$*$trop	dexa$*$gran	dexa$*$drop

Table 5 provides the results of the model selection process for the connected and each disconnected network. After fitting additive CNMA models, we started the model selection by adding a single 2-way interaction. In all disconnected networks, the interaction onda$*$scop minimized *Q* and was selected according to the AIC criterion. The interaction apre$*$scop was selected as the second interaction in the connected network and disconnected networks 1 – 3 while the interaction dexa$*$trop was selected in all other disconnected networks. Similarly, the third interaction was the same in the connected and disconnected networks 1 – 3 (meto$*$trop) and the remaining disconnected networks (dexa$*$gran). In eight disconnected networks a fourth interaction (dexa$*$drop) was also selected.

**Fig. 5:**
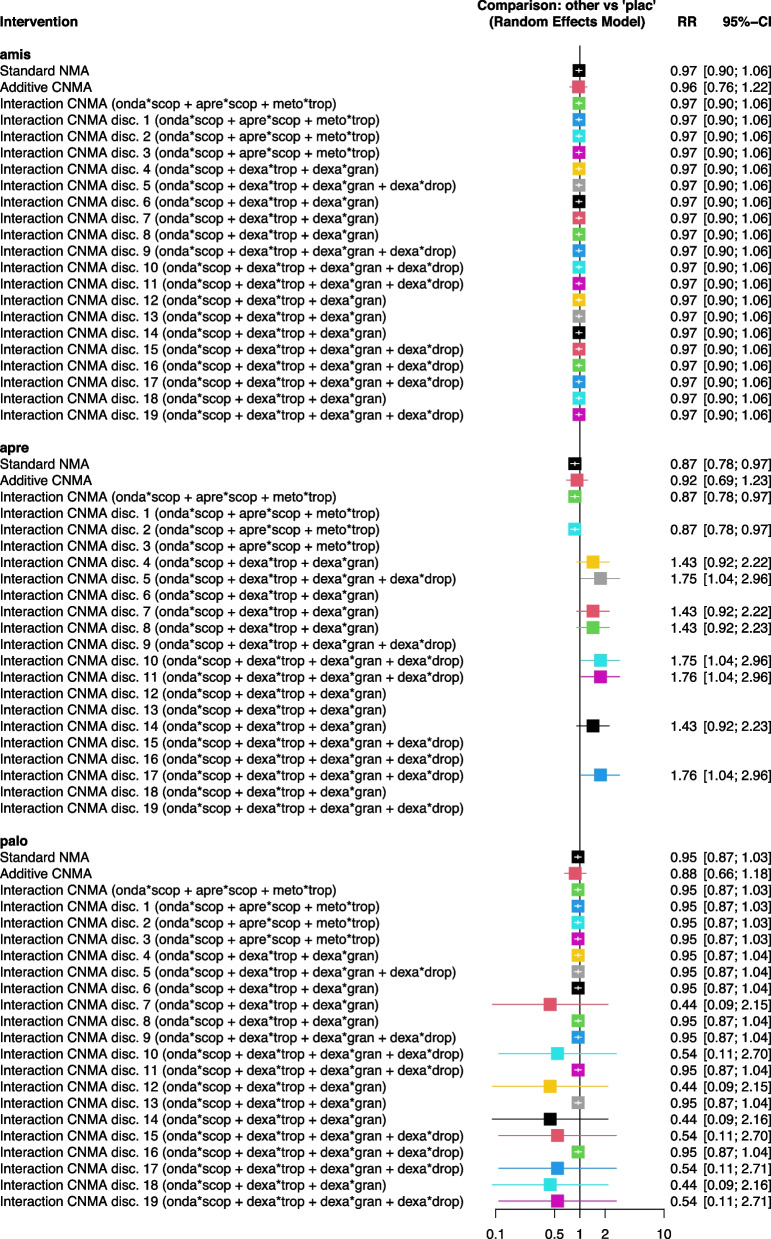
Forest plot of the relative effects of interventions amis, apre, palo with placebo from standard NMA and the selected CNMAs for the connected and each disconnected network

We exemplify the results of the standard NMA and the selected CNMAs of the connected network or disconnected networks by looking at the relative intervention effects of amis, apre and palo compared to placebo (Fig. 5. The full forest plot is provided in the figure in Additional file 8.

(C)NMA results for the comparison amis versus plac are identical for all models (Fig. 5). The intervention amis is only directly compared to plac (Fig. 1) and therefore it is always part of the main subnetwork in disconnected networks with placebo as reference.

The relative intervention effect of apre versus plac is very similar to the standard NMA, the selected CNMA of the connected network and disconnected network 2 (Fig. 5). Otherwise, the estimate of apre versus plac is either inestimable (disconnected networks 1, 3, 6, 9, 12, 13, 15, 16, 18, 19) or misleading (remaining disconnected networks). The effect of apre versus plac is inestimable if the only study comparing apre + scop versus apre constitutes a separate subnetwork (figure in Additional file 7). The component effect apre cancels out due to the additivity assumption, and therefore, the relative intervention effect for apre or apre + scop versus plac is inestimable. For the remaining disconnected networks, the relative intervention effects of apre or apre + scop versus plac are not reliably estimated as intervention apre or apre + scop is not part of the main subnetwork containing plac.

Results for the comparisons palo versus plac are very similar for the standard NMA, selected CNMA of the connected network and some disconnected networks (Fig. 5). Markedly wider confidence intervals are observed for relative effects of palo versus plac in disconnected networks 7, 10, 12, 14, 15, 17 – 19). In these disconnected networks, the intervention palo is not part of the main subnetwork containing plac (figure in Additional file 7).

In general, results of comparisons with placebo are comparable for separate NMA analyses in subnetworks of disconnected networks and the standard NMA in the connected network (figure in Additional file 9). However, comparisons with placebo are only estimable in separate NMAs for treatments in the same subnetwork as placebo. For example, the comparison of apre versus plac is inestimable in all separate NMAs but disconnected network 2 (figure in Additional file 9) as apre is part of an auxiliary network (figure in Additional file 7). On the other hand, the comparison beta versus plac can be estimated in all separate NMA analyses as beta is always part of the main subnetwork.

We conclude that comparisons within the same network are always easier to estimate than comparisons across subnetworks and therefore separate NMA analyses perform better than reconnecting models (here: CNMA models) for disconnected networks.

## Discussion

In this article, we evaluate a model selection strategy for component network meta-analysis that can be used in connected or disconnected networks. In addition, we describe a procedure to create disconnected networks in order to evaluate the properties of the model selection for both connected and disconnected networks. We apply the methods to simulated data and a Cochrane review to investigate their performance.

In connected networks, it is always possible to contrast the results of standard NMA with additive or interaction CNMA models. Accordingly, the application of CNMA models should always be accompanied by a statistical test to assess additivity. We used the difference in *Q* statistics between additive or interaction CNMA and standard NMA model.

The results for the performance of CNMA models are in agreement with the previous simulation study conducted for connected networks by Thorlund and Mills [30]. According to our simulation study, the test for additivity with Q statistic of the difference between the CNMA and NMA model only has sufficient power for a strong violation of additivity. We found that the Q statistic tends to stick to additivity, even if it is strongly violated. As expected, the performance of the model selection procedure for the connected networks deteriorates with increasing heterogeneity. We conclude that the additive CNMA can be an alternative to NMA if the additivity assumption holds and, in general, the selected CNMA and standard NMA provide similar estimates.

The Q statistic of the difference between the CNMA and NMA model is not available for disconnected networks, as standard NMA cannot be fitted. Simulation results are worse for the selected CNMA compared to the additive CNMA for disconnected networks. Our simulation results show that re-connecting disconnected networks with CNMA models is possible only when additivity can be safely assumed. For disconnected networks, we recommend using additive CNMA only if strong clinical arguments for additivity exist. Otherwise, subnetworks should be analyzed separately despite the disadvantage of inestimable treatment comparisons across subnetworks.

The test of additivity was found to have low power in some connected networks which is a general problem of statistical tests based on Cochran’s Q statistics [34, Chapter 10]. For disconnected networks, the additivity assumption cannot be tested. New statistical techniques for the evaluation of additivity assumption are required for both connected and disconnected networks.

Our simulation study shows that CNMA model selection works for connected networks, but not for disconnected networks. Accordingly, we see CNMA model selection as a useful tool for a connected network to evaluate potential interactions between the components of multicomponent interventions. CNMA model selection could in principle be conducted in two directions, forward and backward. As the aim of this simulation study was to evaluate the performance of both connected and disconnected networks, we decided to use only forward selection, to achieve a satisfactory model fit whilst keeping much of the connectivity that is given by the additive model [26]. Forward selection tended to select sparse (often additive) CNMA models for disconnected networks even if additivity was mildly or strongly violated. We only considered 2-way interactions in our simulation, however, the selection procedure could also be used with 3-way or higher interactions.

One limitation of our simulation study is that conclusions depend on the scenarios considered. We simulated a network of interventions with eight interventions and 28 two-arm studies, assuming consistency. We also implemented the forward CNMA model selection process in simulations with the Akaike Information criterion. It is unclear whether different network structures, model assumptions, or a different design or strategy for the model selection process would lead to different conclusions. There is no guarantee that CNMA models behave similarly under different simulation designs.

Another limitation of our simulation study is to only consider the AIC criterion as stopping criterion in the Cochrane data set and simulation study. Use of the AIC criterion results in models with a large number of interaction terms as is visible in the Cochrane data set with up to four 2-way interactions. Several alternative methods are available to conduct model selection. Other methods based on Cochran’s Q statistics like the Bayesian Information Criterion [35] could be easily used instead of the AIC. More advanced methods penalizing the size of the estimated interaction parameters like the LASSO [36] could in principle also be used to select interaction CNMA models, however, this would not be possible using our R scripts.

The application of the additivity test to the Cochrane data set suggests that the additivity assumption does not hold. The network has eleven combinations of two interventions, however, only ten 2-way interactions are estimable. Among the ten 2-way interactions, the interaction onda$*$scop was selected first. The selected CNMA model includes three 2-way interactions (onda$*$scop + apre$*$scop + meto$*$trop) and its results roughly agree with those of the standard NMA. For the disconnected networks in the Cochrane data set, we observed that the estimates can be sometimes similar, sometimes very different, or even inestimable, depending on the network structure. A clinical interpretation of the identified interactions was not the main focus of this work. We think that this would be futile for the general outcome of any adverse events. More specific outcomes should be considered to give a clinical meaning to interactions in CNMA models.

## Conclusions

Although the use of NMA has considerably increased over the last decade, CNMA has not been widely used, but there is an increased clinical interest in the evaluation of multicomponent interventions and we expect an increase in its use. CNMA models are now provided in a frequentist framework, implemented in the R package **netmeta** [33]. This simulation study provides a guidance for CNMA model selection, pointing at some challenges that should be addressed. CNMA models perform well for connected networks, and can be an alternative to standard NMA if additivity holds. On the contrary, CNMA models do not perform well for disconnected networks, and we recommend conducting separate analyses of subnetworks. In conclusion, we advise the usage of CNMA methods for connected networks when multicomponent interventions are evaluated in practice, but caution is needed with CNMA methods in case of disconnected networks.

## Supplementary Information

**Additional file 1.**

**Additional file 2.**

**Additional file 3.**

**Additional file 4.**

**Additional file 5.**

**Additional file 6.**

**Additional file 7.**

**Additional file 8.**

**Additional file 9.**

**Additional file 10.**

## Data Availability

The data set analysed in the current study and the programming code are publicly available on https://zenodo.org/badge/latestdoi/546041022.

## Abbreviations

amis: Amisulpride
apre: Aprepitant
beta: Betamethasone
caso: Casopitant
CNMA: Component network meta-analysis
CP: Coverage probability
dexa: Dexamethasone
dime: Dimenhydrinate
dola: Dolasetron
drop: Droperidol
gran: Granisetron
meto: Metoclopramide
MSE: Mean squared error
NMA: Network meta-analysis
OR: Odds ratio
onda: Ondansetron
palo: Palonosetron
plac: Placebo
ramo: Ramosetron
RCT: Randomized controlled trial
RR: Risk ratio
scop: Scopolamine
trop: Tropisetron
vest: Vestipitant
