# Supplementary material for: Model selection for component network meta-analysis in connected and disconnected networks: a simulation study
Source: BMC Med Res Methodol. 2023 Jun 14;23:140. doi: 10.1186/s12874-023-01959-9 (PMC10268445; doi:10.1186/s12874-023-01959-9)
Supplement: Supplementary file 1 — Additional file 1. [file 12874_2023_1959_MOESM1_ESM.pdf]

Intervention	Abbreviation	Addition
amisulpride	amis	step 2
betamethasone	beta	step 2
dexamethasone	dexa	step 4
dexamethasone+droperidol	dexa+drop	step 4
dexamethasone+ondansetron	dexa+onda	step 4
dexamethasone+granisetron	dexa+gran	step 5
dexamethasone+tropisetron	dexa+trop	step 4
dolasetron	dola	step 2
droperidol	drop	step 4
droperidol+granisetron	drop+gran	step 4
granisetron	gran	step 4
metoclopramide+scopolamine	meto+scop	step 3
placebo	plac	step 1
scopolamine	scop	step 3
tropisetron	trop	step 5
