# Supplementary figures and images for: Model selection for component network meta-analysis in connected and disconnected networks: a simulation study
Source: BMC Med Res Methodol. 2023 Jun 14;23:140. doi: 10.1186/s12874-023-01959-9 (PMC10268445; doi:10.1186/s12874-023-01959-9)

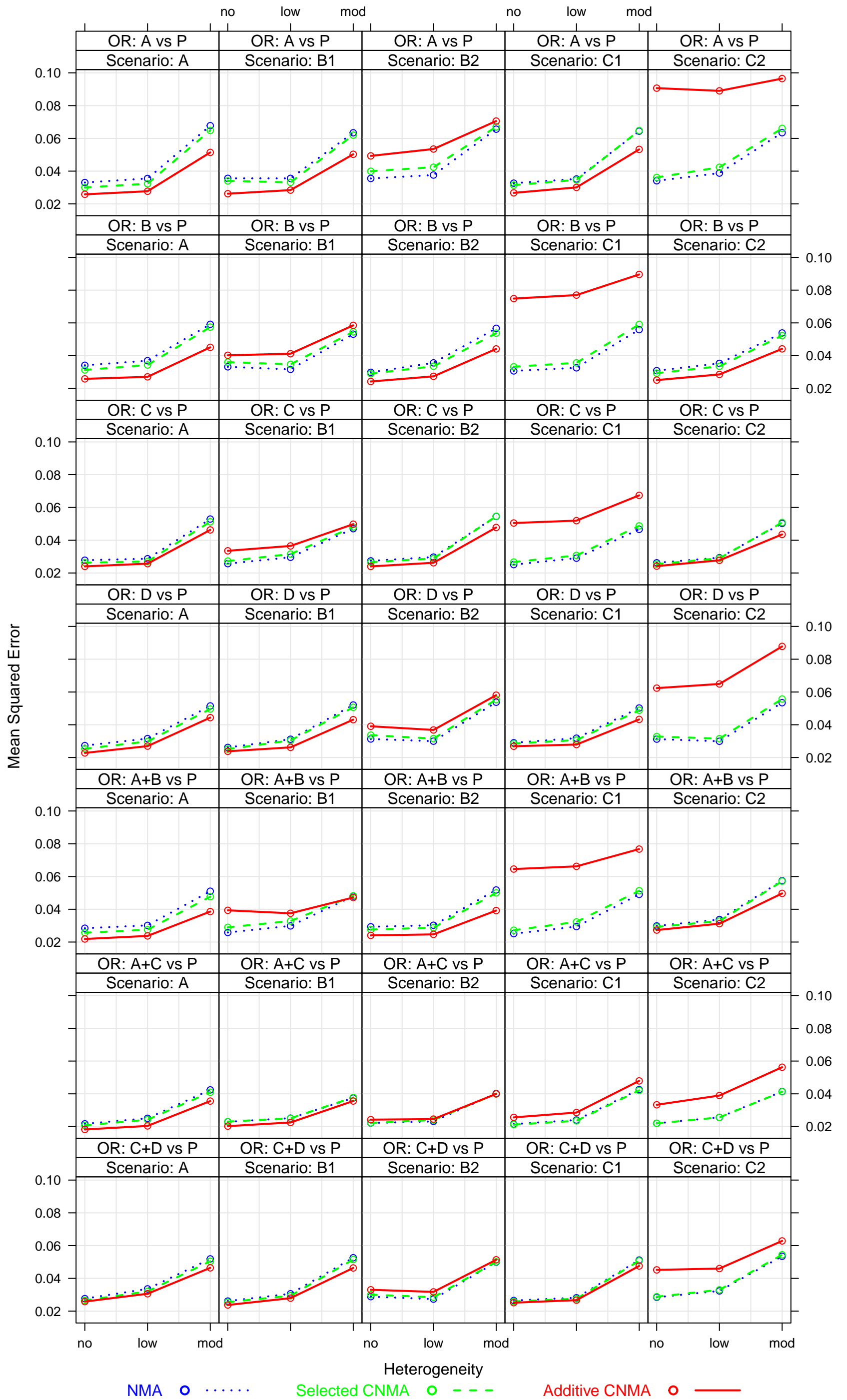

Supplement: Supplementary file 2 — Additional file 2. [file 12874_2023_1959_MOESM2_ESM.pdf]

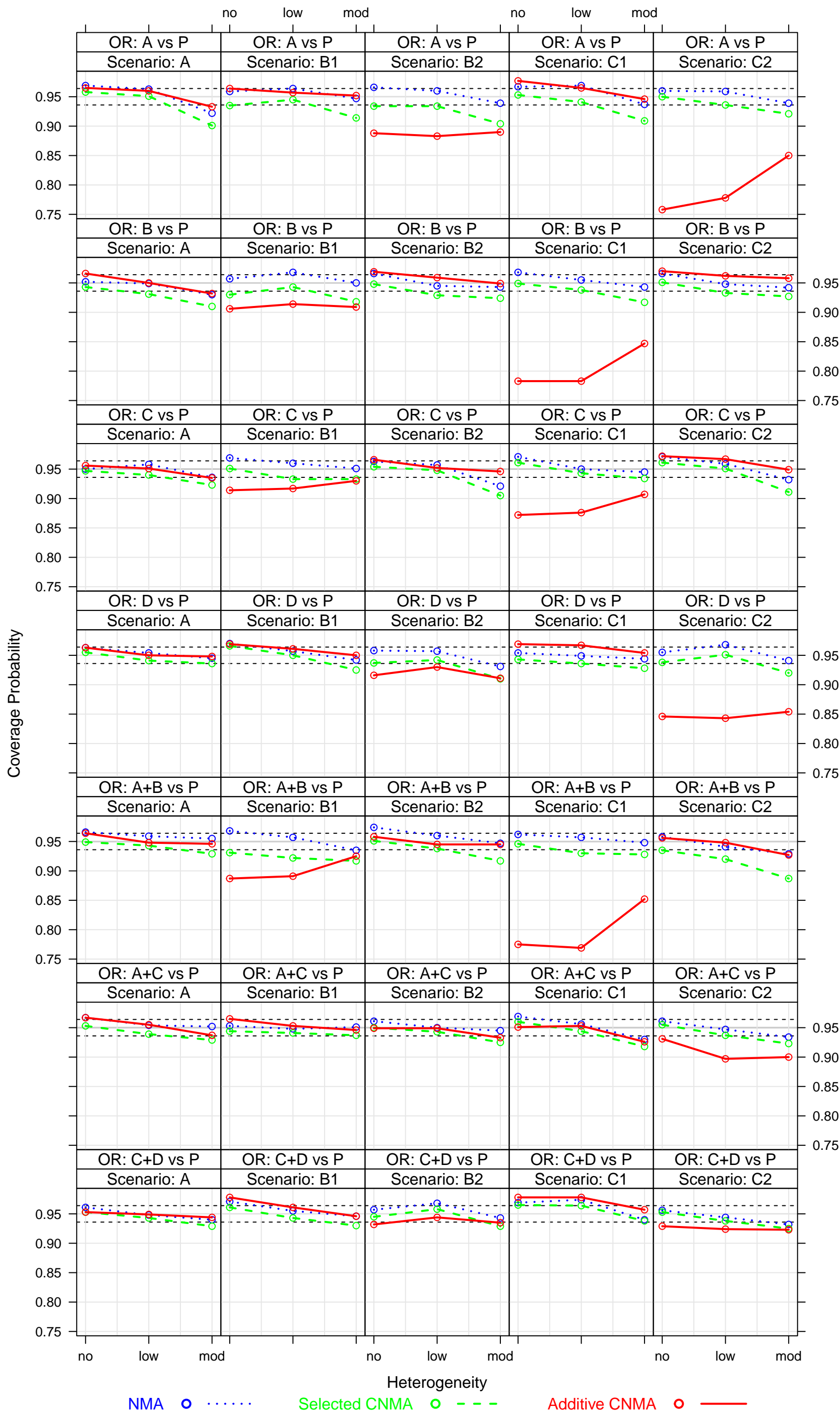

Supplement: Supplementary file 3 — Additional file 3. [file 12874_2023_1959_MOESM3_ESM.pdf]

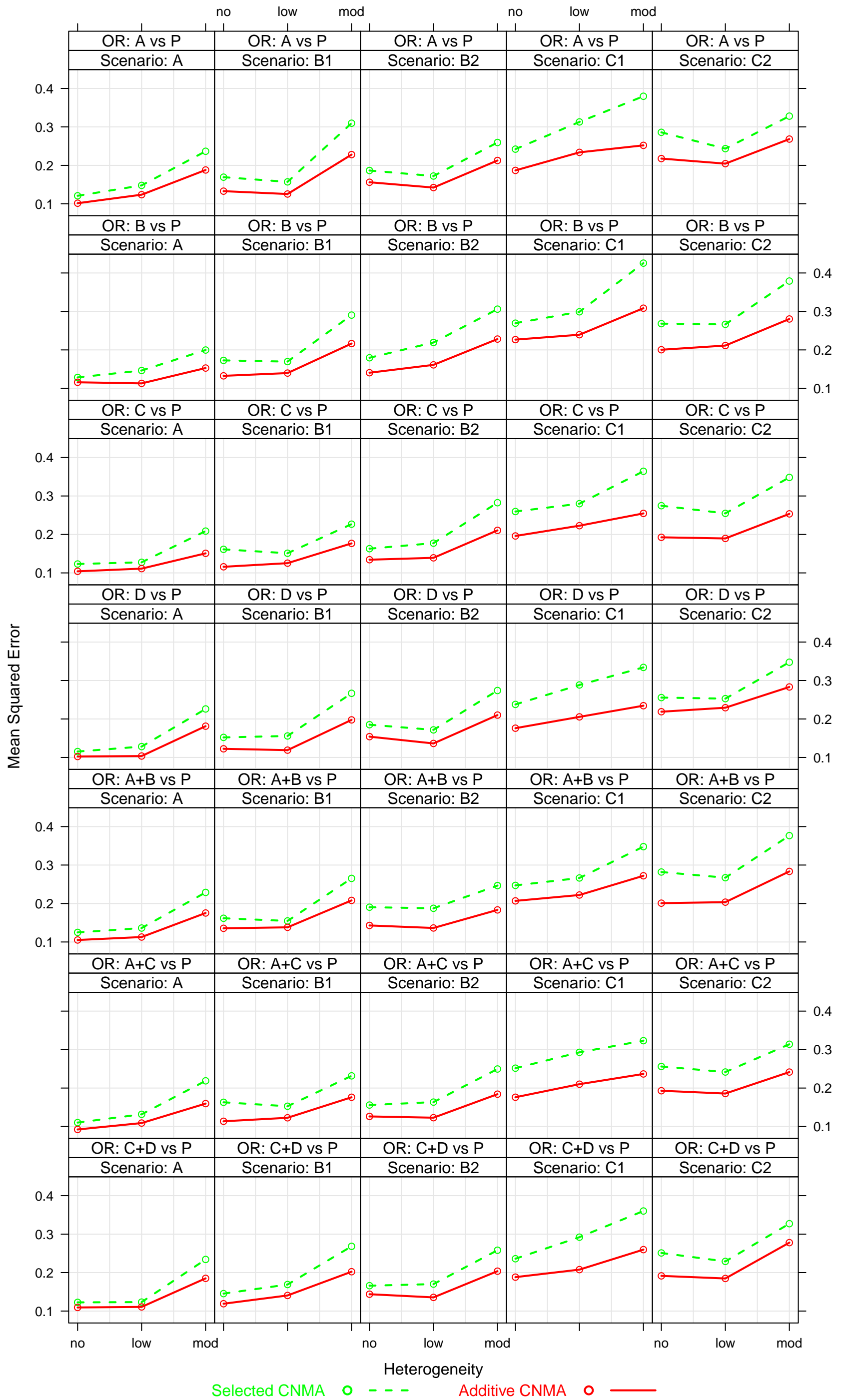

Supplement: Supplementary file 4 — Additional file 4. [file 12874_2023_1959_MOESM4_ESM.pdf]

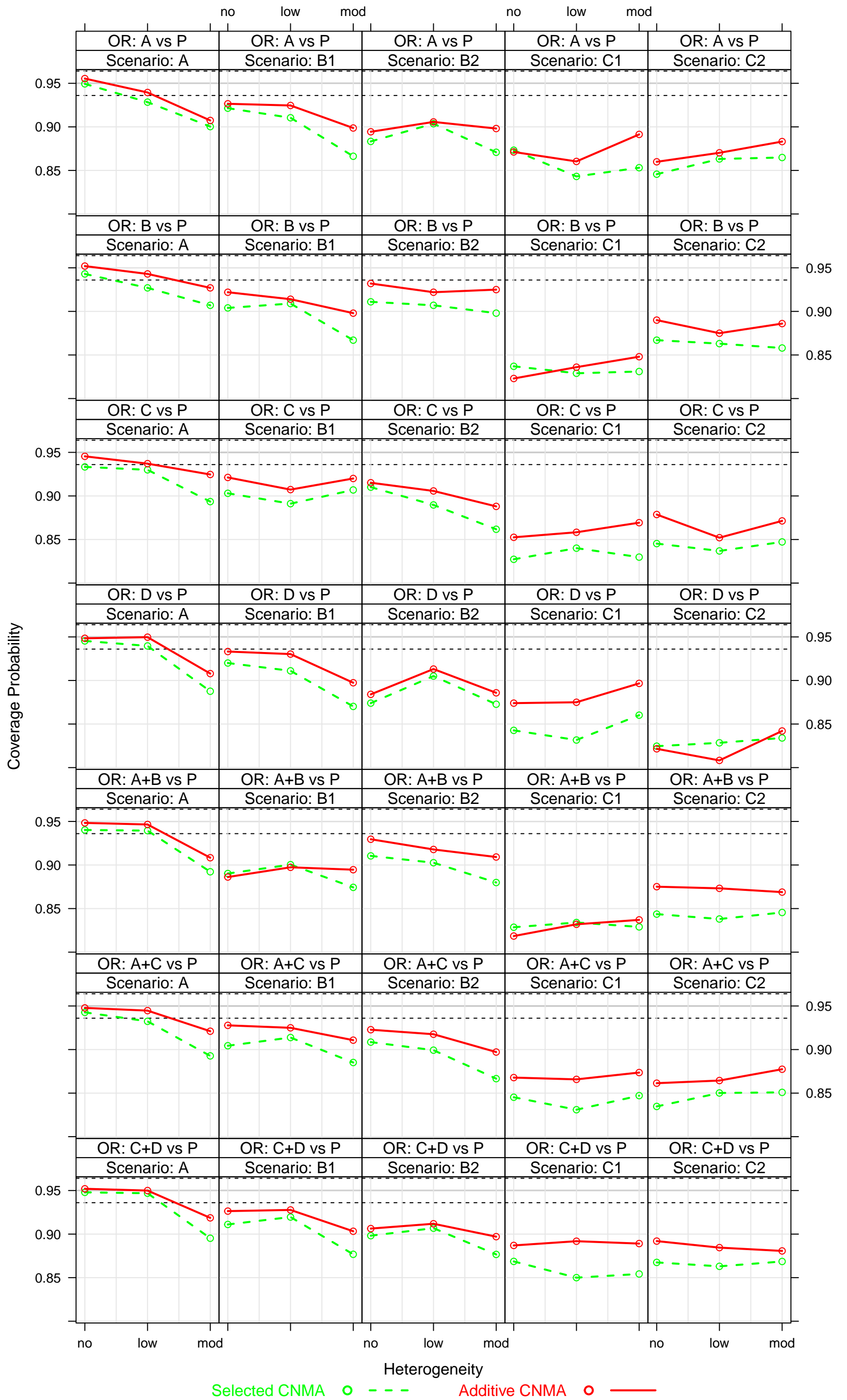

Supplement: Supplementary file 5 — Additional file 5. [file 12874_2023_1959_MOESM5_ESM.pdf]

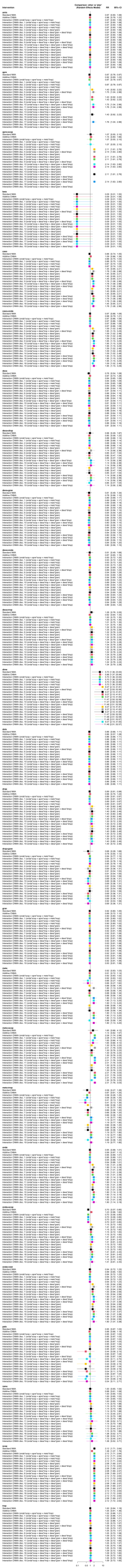

Supplement: Supplementary file 8 — Additional file 8. [file 12874_2023_1959_MOESM8_ESM.pdf]

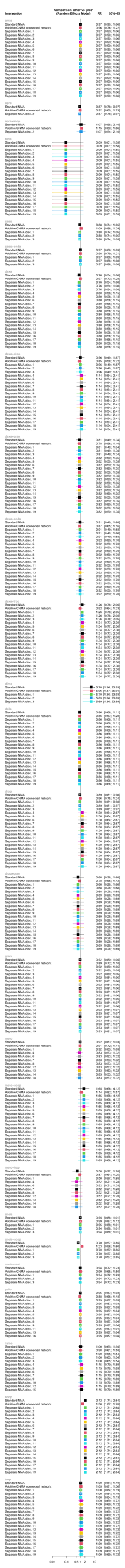

Supplement: Supplementary file 9 — Additional file 9. [file 12874_2023_1959_MOESM9_ESM.pdf]
