# Supplementary material for: Model selection for component network meta-analysis in connected and disconnected networks: a simulation study
Source: BMC Med Res Methodol. 2023 Jun 14;23:140. doi: 10.1186/s12874-023-01959-9 (PMC10268445; doi:10.1186/s12874-023-01959-9)
Supplement: Supplementary file 6 — Additional file 6. [file 12874_2023_1959_MOESM6_ESM.pdf]

Additional file 6 - additional results for Cochrane review data set

A standard NMA of the Cochrane data set does not show any substantial between-study heterogeneity / inconsistency ($Q = 44.80, df = 46, p = 0.5227$). In the additive CNMA model which estimates 17 component effects (including placebo) the between-study heterogeneity / inconsistency is much larger ($Q = 103.53, df = 55, p < 0.0001$). The difference in Q statistics between additive CNMA and standard NMA is highly statistical significant ($Q_{diff} = 58.74, df = 9, p < 0.0001$), showing that the additivity assumption is not justified for all observed treatment combinations. Accordingly, we used the forward CNMA model selection procedure to add interaction terms and to relax the additivity assumption.

The network has 11 combinations of two interventions, however, one component (vest) was only evaluated in the combination onda + vest. The interaction onda∗vest cannot be distinguished from the effect of vest, leaving ten potential 2-way interaction terms. Starting from the additive CNMA model, we added a single 2-way interaction term to the model (table in additional file 10). All ten interaction models led to a reduction of Q with the largest reduction for the interaction onda∗scop ($Q = 53.70, df = 54, p = 0.4860$). This interaction CNMA model is preferred over the additive CNMA model according to the AIC criterion ($p < 0.0001$).

In the next selection step, all 45 combinations of two 2-way interactions were considered. Only nine models with two 2-way interactions led to a further reduction of Q (table in additional file 10). All nine models included the interaction onda∗scop selected in the first step plus one additional interaction term. The largest reduction of Q was observed for the interactions onda∗scop + apre∗scop ($Q = 50.19, df = 53, p = 0.5841$). This model was preferable to the CNMA model with one 2-way interaction according to the AIC criterion ($p = 0.0611$).

In total, ten of 120 combinations of three 2-way interactions further decreased Q. All ten models included the interaction onda∗scop, however, only 8 of 10 models included the interaction apre∗scop which was selected in the second step. Three of the 10 models included the interaction meto∗trop which was also part of the model with the smallest heterogeneity/inconsistency: onda∗scop + apre∗scop + meto∗trop ($Q = 47.71, df = 52, p = 0.6432$). Based on the AIC criterion, this combination of three 2-way CNMA interactions was preferable to a model with two 2-way interactions ($p = 0.1197$). Overall, the results of this selected interaction CNMA model are very similar to the standard NMA (additional file 8). Only for the combination meto + scop versus plac, the standard NMA reports a non-significant result (RR = 1.65 [0.66; 4.12]) while the selected interaction CNMA model estimates a stronger, significant effect (RR = 2.28 [1.74; 2.99]). In contrast,

results for the additive CNMA are different for several comparisons, especially, onda + scop vs plac or scop vs plac (additional file 8).
