# Supplementary material for: Model selection for component network meta-analysis in connected and disconnected networks: a simulation study
Source: BMC Med Res Methodol. 2023 Jun 14;23:140. doi: 10.1186/s12874-023-01959-9 (PMC10268445; doi:10.1186/s12874-023-01959-9)
Supplement: Supplementary file 7 — Additional file 7. [file 12874_2023_1959_MOESM7_ESM.pdf]

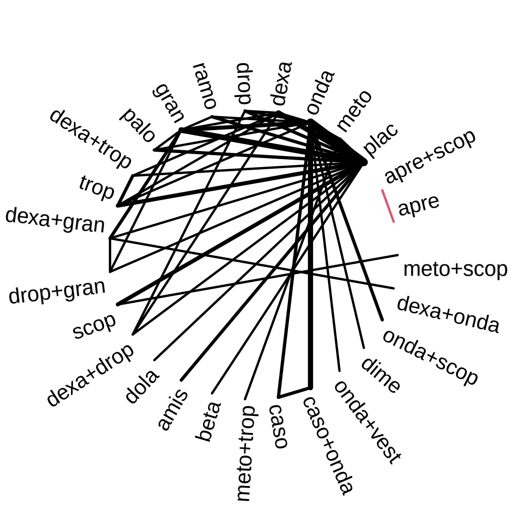

Disconnected 1 (m=87, k=55)

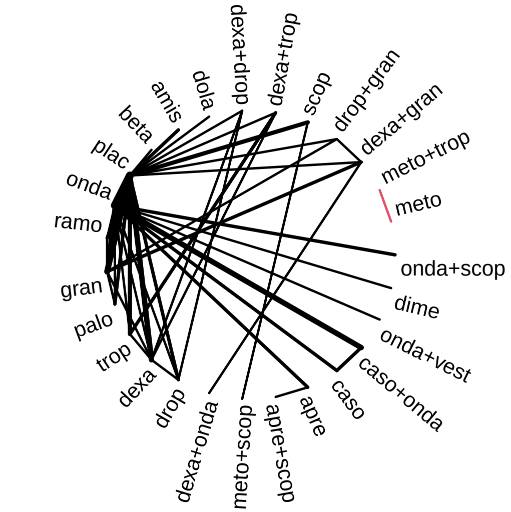

Disconnected 2 (m=83, k=53)

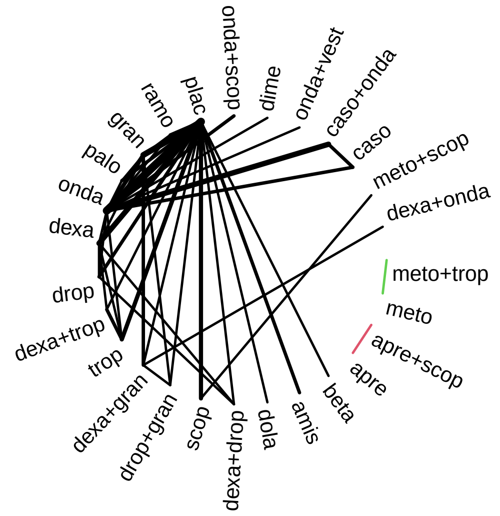

Disconnected 3 (m=81, k=51)

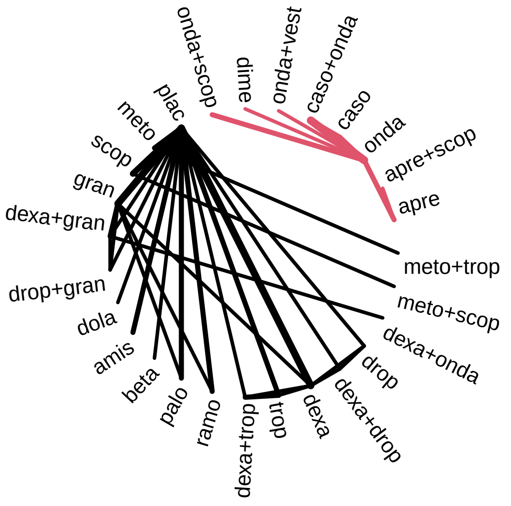

Disconnected 4 (m=61, k=41)

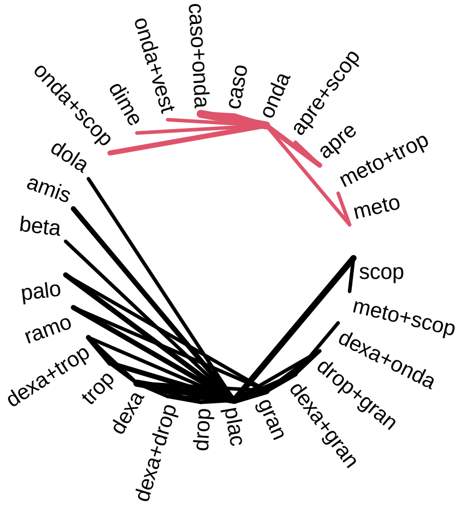

Disconnected 5 (m=60, k=40)

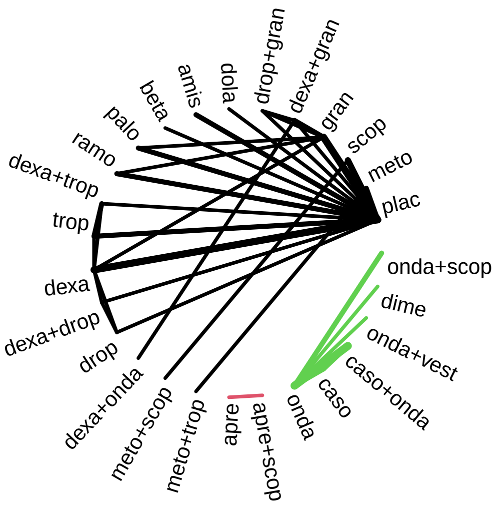

Disconnected 6 (m=59, k=39)

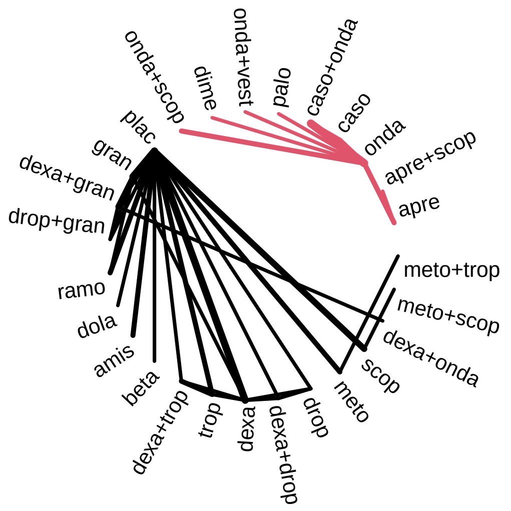

Disconnected 7 (m=59, k=39)

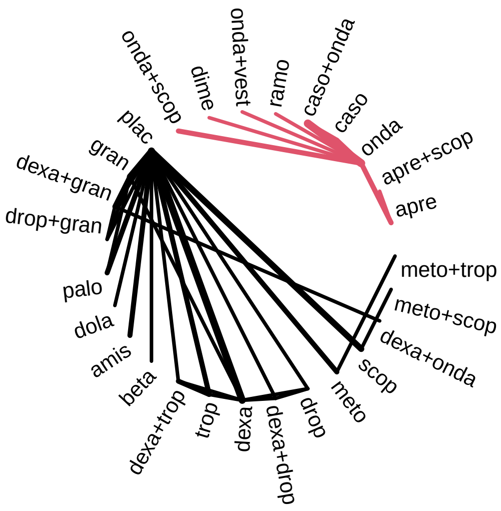

Disconnected 8 (m=58, k=40)

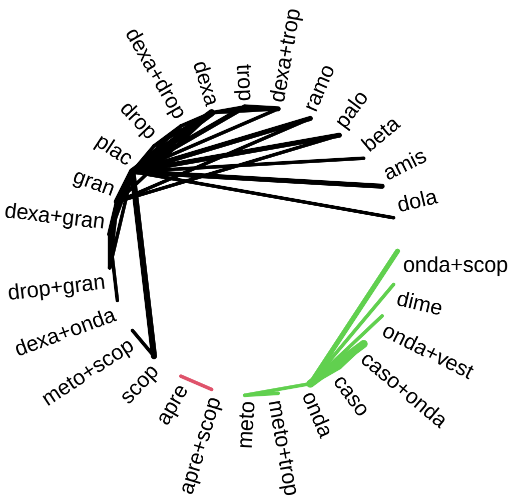

Disconnected 9 (m=58, k=38)

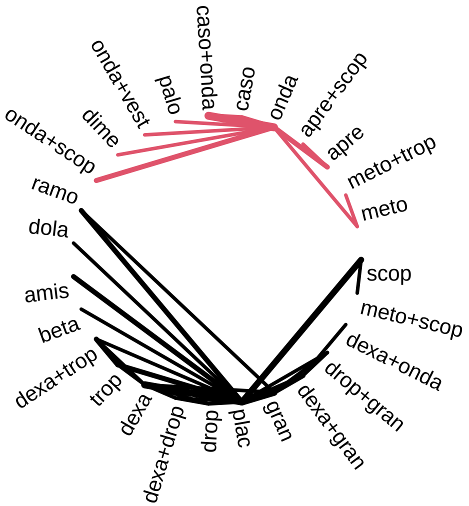

Disconnected 10 (m=58, k=38)

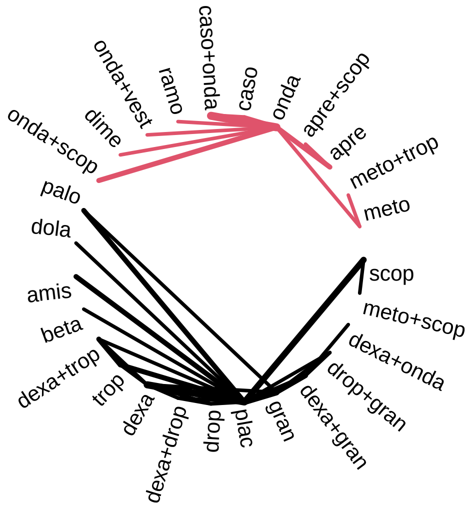

Disconnected 11 (m=57, k=39)

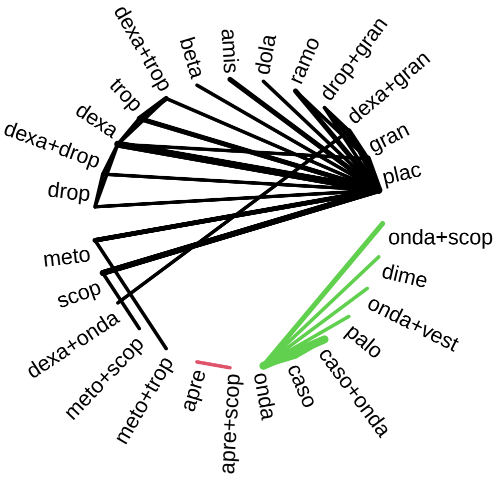

Disconnected 12 (m=57, k=37)

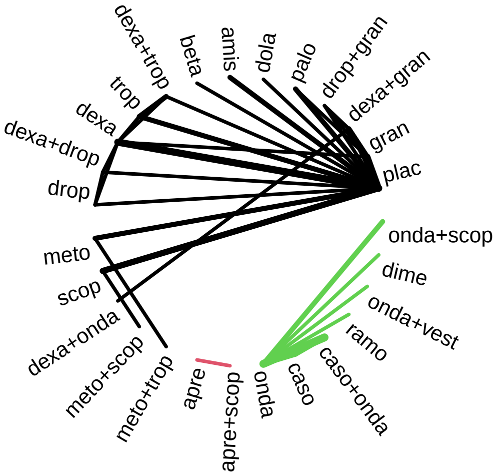

Disconnected 13 (m=56, k=38)

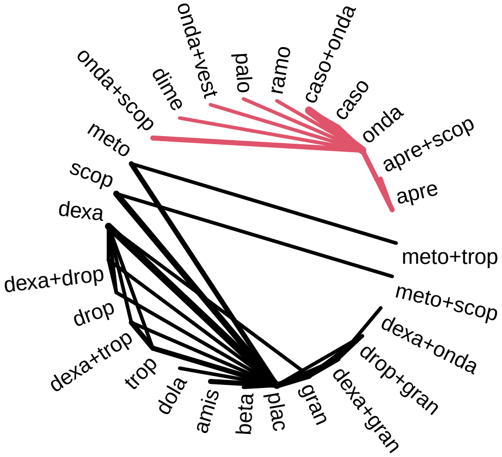

Disconnected 14 (m=56, k=38)

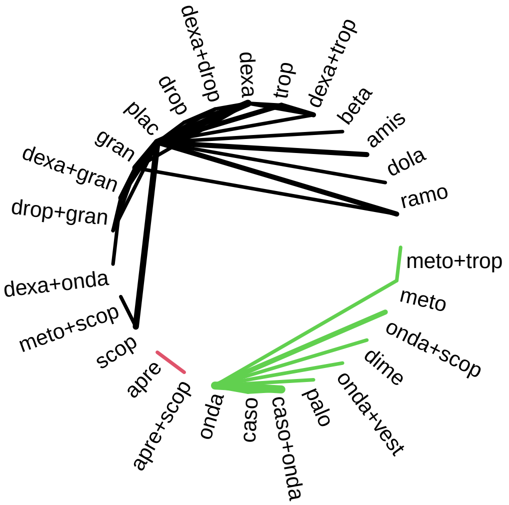

Disconnected 15 (m=56, k=36)

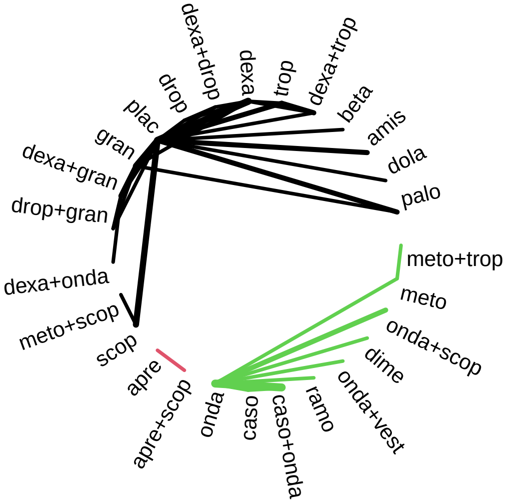

Disconnected 16 (m=55, k=37)

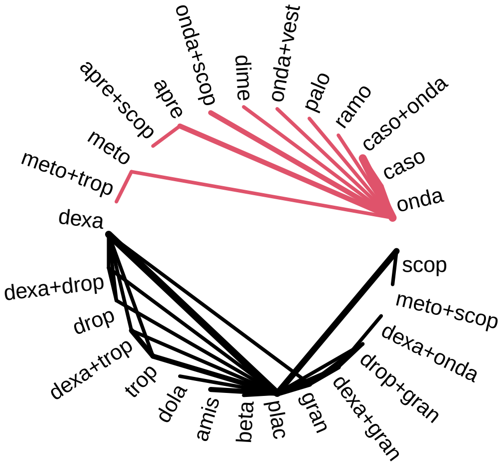

Disconnected 17 (m=55, k=37)

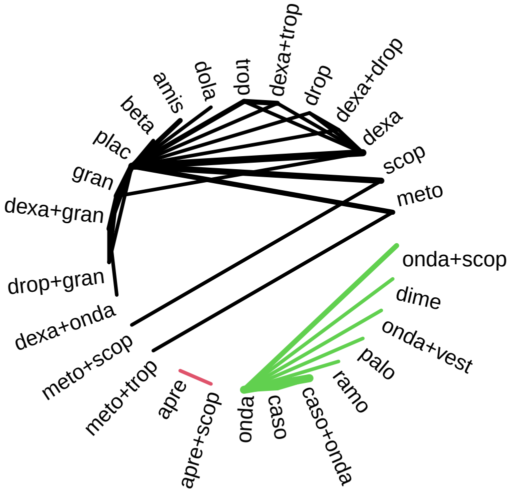

Disconnected 18 (m=54, k=36)

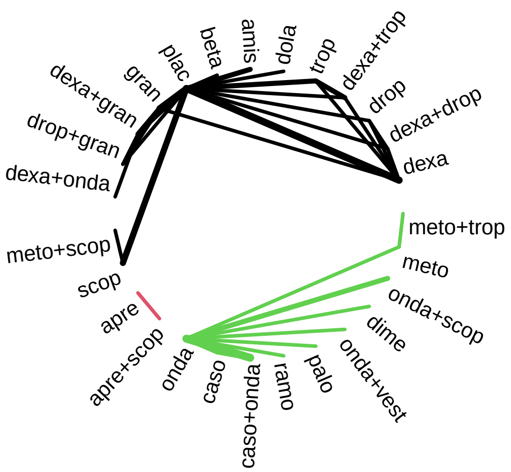

Disconnected 19 (m=53, k=35)
