# Supplementary material for: Model selection for component network meta-analysis in connected and disconnected networks: a simulation study
Source: BMC Med Res Methodol. 2023 Jun 14;23:140. doi: 10.1186/s12874-023-01959-9 (PMC10268445; doi:10.1186/s12874-023-01959-9)
Supplement: Supplementary file 10 — Additional file 10. [file 12874_2023_1959_MOESM10_ESM.pdf]

Standard NMA

Interaction term	Heterogeneity		
	Q	df	p-value
Full interaction model	44.80	46	0.5227

Additive CNMA

Interaction term	Heterogeneity			Difference[1]
	Q	df	p-value	p-value
No interaction	103.53	55	< 0.0001	< 0.0001

Interaction CNMA (one 2-way interaction)

Interaction term	Heterogeneity			Difference[2]
	Q	df	p-value	p-value
dexa∗gran	103.52	54	0.0001	0.9072
dexa∗onda	103.49	54	0.0001	0.8295
drop∗gran	103.48	54	0.0001	0.8201
meto∗scop	103.48	54	0.0001	0.8141
apre∗scop	103.42	54	0.0001	0.7383
dexa∗drop	102.91	54	0.0001	0.4294
meto∗trop	102.47	54	0.0001	0.3025
dexa∗trop	100.12	54	0.0001	0.0647
caso∗onda	76.53	54	0.0235	< 0.0001
onda∗scop⋆	53.70	54	0.4859	< 0.0001

Interaction CNMA (two 2-way interactions)

Interaction term	Heterogeneity			Difference[3]
	Q	df	p-value	p-value
onda∗scop + dexa∗onda	53.54	53	0.0885	0.6913
onda∗scop + dexa∗drop	53.52	53	0.4533	0.6672
onda∗scop + dexa∗gran	53.46	53	0.4544	0.6263
onda∗scop + drop∗gran	53.35	53	0.4564	0.5550
onda∗scop + meto∗scop	53.30	53	0.4606	0.5255
onda∗scop + caso∗onda	52.55	53	0.4627	0.2846
onda∗scop + dexa∗trop	51.83	53	0.4914	0.1719
onda∗scop + meto∗trop	51.41	53	0.5196	0.1303
onda∗scop + apre∗scop⋆	50.19	53	0.5362	0.0611

Interaction CNMA (three 2-way interactions)

Interaction term	Heterogeneity			Difference[4]
	Q	df	p-value	p-value
onda∗scop + apre∗scop + caso∗onda	50.05	52	0.5508	0.7070
onda∗scop + apre∗scop + dexa∗drop	50.05	52	0.5510	0.7018
onda∗scop + apre∗scop + dexa∗onda	50.02	52	0.5523	0.6738

onda∗scop + apre∗scop + dexa∗gran	49.91	52	0.5566	0.5923
onda∗scop + apre∗scop + drop∗gran	49.79	52	0.5614	0.5236
onda∗scop + dexa∗trop + meto∗trop	49.79	52	0.5614	0.5232
onda∗scop + apre∗scop + meto∗scop	49.72	52	0.5640	0.4919
onda∗scop + caso∗onda + dexa∗trop	49.26	52	0.5821	0.3350
onda∗scop + apre∗scop + dexa∗trop	48.49	52	0.6125	0.1924
onda∗scop + apre∗scop + meto∗trop⋆	47.71	52	0.6432	0.1149

[1]Difference to standard NMA model; [2]difference to additive CNMA model; [3]difference to selected interaction CNMA with one 2-way interaction; [4]difference to selected interaction CNMA with two 2-way interactions; ⋆selected interaction CNMA model with one, two or three 2-way interactions
